# Supplementary material for: The causal relationship between the human gut microbiota and pyogenic arthritis: a Mendelian randomization study
Source: Front Cell Infect Microbiol. 2024 Nov 26;14:1452480. doi: 10.3389/fcimb.2024.1452480 (PMC11629706; doi:10.3389/fcimb.2024.1452480)
Supplement: Supplementary file 1 [file DataSheet1.zip › mendelian test/ebi-a-GCST90027593.csv_forest.pdf]

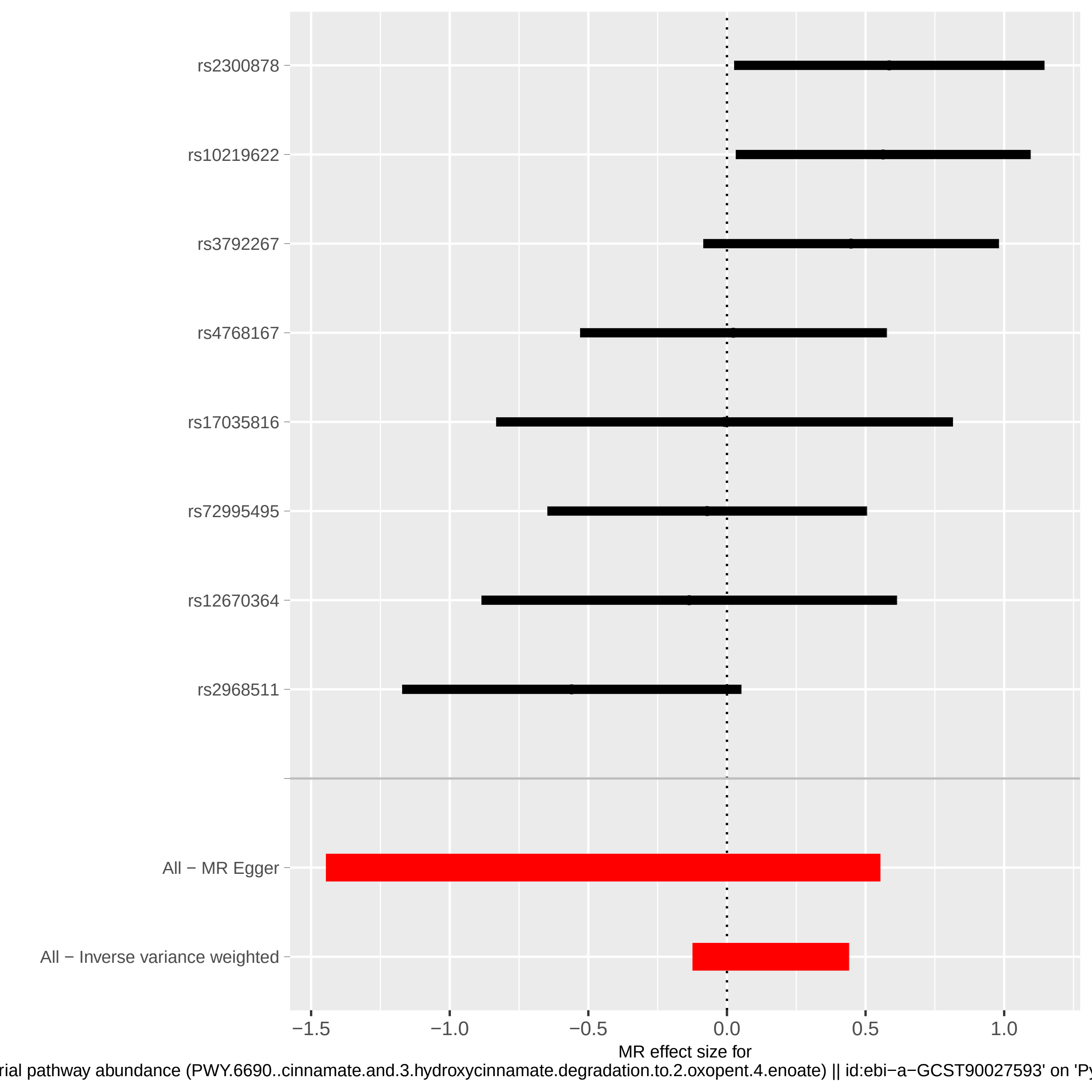

rs2300878
rs10219622
rs3792267
rs4768167
rs17035816
rs72995495
rs12670364
rs2968511
All − MR Egger
All − Inverse variance weighted
−1.5
−1.0
−0.5
0.0
0.5
1.0
MR effect size for
rial pathway abundance (PWY.6690..cinnamate.and.3.hydroxycinnamate.degradation.to.2.oxopent.4.enoate) || id:ebi−a−GCST90027593' on 'Py
